# Instances of Biowarfare in World War I (1914–1918)

**DOI:** 10.7759/cureus.59329

**Published:** 2024-04-29

**Authors:** Ioannis Nikolakakis, Spyros N Michaleas, George Panayiotakopoulos, Theodore G Papaioannou, Marianna Karamanou

**Affiliations:** 1 Emergency Department, Tzaneio Prefecture General Hospital of Piraeus, Athens, GRC; 2 Department of History of Medicine and Medical Ethics, National and Kapodistrian University of Athens School of Medicine, Athens, GRC; 3 Department of Pharmacology, School of Medicine, University of Patras, Patras, GRC

**Keywords:** biological agents, sabotage, anthrax, glanders, bioweapon

## Abstract

During World War I (WWI), also referred to as 'The Great War,' Germany implemented a pioneering biowarfare program as part of a broader military strategy to undermine Allied forces by targeting their logistical and supply capabilities. This initiative, unprecedented in its systematic and strategic application, utilized a variety of pathogens, primarily targeting animal populations, to disrupt support systems without contravening international laws, specifically the 1907 Hague Convention. The operations, shrouded in secrecy and largely led by the German General Staff, included sophisticated sabotage actions against both enemy and neutral states. The allegations and usage of bioweapons increased the interest of the Great Powers in further developing their own biowarfare program.

## Introduction and background

World War I (WWI), often referred to as "The Great War" or optimistically as "The War to End All Wars," was widely anticipated by many around the globe to bring an end to international conflicts, sealing a promise of lasting peace. However, contrary to these hopes, it only laid the groundwork for further global turmoil, eventually leading to an even more devastating conflict. During this tumultuous period, Germany embarked on a groundbreaking and ethically contentious endeavor: the use of biological warfare. This type of warfare, executed on a scale and with a level of sophistication previously unseen, was embodied in Germany's broader military strategy.

The core objective of Germany's biowarfare program was to severely impair the logistical support and supply chains of the Allied forces, thus weakening their overall war efforts. To achieve this, the German military, under the directive of the General Staff, employed a variety of pathogenic agents. According to the rationale of the German military leadership, their tactics did not directly contravene the established rules of war since they were aimed at undermining the material capabilities of their adversaries rather than harming soldiers directly, thereby sidestepping clear violations of contemporary international laws and instead targeting the animals that were critical to the support structures of the military and essential for transportation and communication.

This calculated approach allowed Germany to maintain adherence to the legal standards of warfare as established by the 1907 Hague Convention [1]. This nuanced interpretation of the laws of war facilitated a campaign that was extensive and clandestinely executed against not only enemy states but also neutral countries that might have been indirectly supporting Allied logistics. Nations such as Romania, the United States, Spain, Norway, and Argentina found themselves targets of these covert operations.

The history of glanders prior to WWI

Glanders, caused by the Gram-negative bacterium *Burkholderia mallei*, is a contagious and zoonotic disease affecting solipeds. The sole natural hosts for* B. mallei *are equines such as horses, donkeys, and mules. The disease is marked by ulcerative nodular lesions on the skin and mucous membranes at the site of the infection, which might be a scratch or a cut. The dissemination of the disease might occur one to four weeks after the initial infection. Broad symptoms consist of fever, general discomfort, muscle aches, chest pain, headache, and nasal discharge. The disease usually progresses to pulmonary infection and septic shock [2]. If left untreated, glanders has a mortality rate of 90%, but infection in humans has been a rare occurrence [3].

Glanders is one of the oldest recorded infectious diseases, with symptoms documented by Hippocrates as early as 425 BC. Aristotle described the disease in horses in 330 BC and named it "malleus" [4]. The disease was associated with the gathering of horses in various parts of the world, particularly military horses and mules. By the 4th century, the Byzantine veterinarian Absyrtus and the scholar Vegetius recognized the infectious nature of the disease and recommended isolating affected animals [5]. Glanders was not systematically studied until several centuries later. The first veterinary school was established in Lyon, France, in the mid-18th century to address the serious problems of glanders. In 1837, Rayer demonstrated the disease's transmissibility by infecting a horse using purulent fluid from an infected animal [6]. In 1882, Loeffler and Schütz isolated the causative agent, *B. mallei*, in cultures from the liver and spleen of a horse [7]. The problem worsened after the American Civil War, when military animals were sold to civilians, facilitating the spread of the disease to communities [8].

In the late 19th century, several countries initiated control and eradication programs. The initial programs included only the culling of clinically ill equines and careful disinfection of the premises. Despite these tactics, glanders would reappear in new or remaining animals in stables and barns that once housed infected animals, and the number of cases across the US increased without the occurrence of human epidemics. Vaccines and therapeutic agents were developed with little success, and by 1890, the diagnostic mallein skin test was developed [9]. Control and eradication programs would soon include testing all contact equines, followed by quarantine and recommendation for the culling of all animals with a positive test. This program initially failed in some locations due to a lack of enforcement and incentives for owners to cull their non-clinically ill animals. Some equine owners would intentionally hide their animals to avoid testing or would repeatedly sell asymptomatic animals with a positive test to avoid financial losses.

The discovery of the steam engine further helped spread the disease by transporting *B. mallei*-infected animals to other areas and countries. Once the owners were offered compensation for animals with positive mallein tests, an increase was seen in the disease control of glanders, which was finally eradicated in Great Britain by 1928, in Canada by 1938, and in the USA by 1942, about 30 years after the start of the programs [10]. Glanders would serve as a primary biological weapon until the mechanization of armies, which eventually led to the replacement of animals, consequently reducing its significance.

Anthrax before WWI

The zoonotic disease of anthrax, caused by *Bacillus anthracis*, is a gram-positive, spore-forming *Bacillus* that affects animals and humans via contact with infected animals or their products. Anthrax presents in three forms: cutaneous, intestinal, and inhalational [11]. The most common, cutaneous anthrax, features skin ulcers with a black center and typically has a low mortality rate with treatment. Intestinal anthrax results from eating contaminated meat, leading to severe gastrointestinal issues, while inhalational anthrax, associated with spore inhalation, can progress rapidly to severe respiratory and systemic issues if untreated.

Inhalational anthrax, contracted from breathing in spores from animal products or during bioattacks, initially affects the lungs. The spores are engulfed by macrophages and transported to lymph nodes, releasing toxins that may lead to severe lung and systemic infections. This form has a two-phase progression: an initial phase with general symptoms like muscle aches, fever, and tiredness, followed by a critical phase of severe breathing difficulties and shock, often fatal without antibiotics [12].

Anthrax is strongly linked to the fifth and sixth plagues of Moses in the Bible, which describe the mass death of cattle, camels, sheep, and other animals, followed by the sixth plague initiated during the collection of the dead animals. The Egyptians likely contracted cutaneous anthrax during this process [13]. Anthrax was first noted by Virgil in Georgics, and later in 1727, Nicolas Fournier (1700-1781) identified two forms: charbon spontané, thought to arise from sun exposure, and charbon contagieux, contracted from infected meat or animal products. Robert Koch (1843-1910) discovered the bacterium in 1875. Its use as a biological warfare agent began during World War I, and post-war, it was extensively developed in the Soviet Union [14].

## Review

Materials and methods

A comprehensive literature search was conducted to identify relevant studies and information related to the use of bioweapons during WWI. Secondary sources, including peer-reviewed journal articles, review papers, and relevant textbooks, were utilized for this purpose. To complement the sources, additional bibliography was gathered from recent book editions analyzing primary sources, historical documents, and archives.

The biosabotage of Otto von Rosen

On a state level, Germany was a pioneer of both biological and chemical warfare during WW, while the Entente nations, namely France, Russia, and Great Britain, invested mostly in chemical warfare. The German General Staff led an ambitious biological warfare campaign, primarily targeting military animals rather than enemy soldiers, essentially conducting "biological sabotage," and concluded that the use of these agents did not violate the 1907 Hague Convention. These activities, mostly revealed through British espionage and cryptography by Room OB40 and confirmed by British agents and authorities of the targeted countries, involved executing biological attacks aimed at disrupting supply chains and the conduct of the war [15]. These operations aimed to cripple the supply chains that were crucial for the Allies' war efforts. A Swedish army officer and nobleman, Baron Otto Karl Robert von Rosen (1884-1963), during WWI, was a central figure in a series of plans involving biological warfare and sabotage actions against Russian troops in Finland and surrounding areas. One effort was to infect the reindeer used to transport British weapons through northern Norway and to equip Finns with anthrax vials [16,17]. Before the development of an effective anthrax vaccine, the disease caused the deaths of millions of cattle globally.

In 1916, Norwegian counterintelligence began to take an interest in von Rosen, despite Norway's neutrality during the war, as he seemed to be planning more than mere commercial activities. It was confirmed via message exchanges revealed by counterintelligence that Von Rosen was involved in a covert mission that included the risky transport of anonymous material from Denmark to Sweden. His activities quickly expanded to include biological and unconventional warfare against the Russians in Finland, aimed at undermining the Tsar's control of the area. From Germany, von Rosen was sent vials with anthrax spores, which were placed in the feed of Russian stables, intended to cause disease in military horses. Returning to Stockholm in September 1916, von Rosen and his associates transported explosives and other supplies to northern Sweden, disguised as canned meat. When local police arrested them, they managed to convince them that they were transporting medicines for Finnish revolutionaries, but the true nature of the 'medicines' as explosives was eventually revealed.

At first glance, the equipment's cargo did not contain a large volume of explosives, and some additional items, such as a camera, a pistol, a rifle, maps of Russia and Finland, von Rosen's diary, a bottle of oral solution, a small vial, two boxes of sugar cubes, and coins. His associates revealed during interrogations that their mission aimed to stir anti-Russian sentiment in Finland and support its independence, with von Rosen confirming that the purpose of the mission was to assist the Finnish revolutionaries. However, there was no mention of anthrax. Despite Norway's neutrality, public opinion sympathized with the Finnish revolutionaries, leading Norwegian politicians to seek a quick settlement of the case [18]. Twenty-one days after his arrest, Von Rosen was expelled to Sweden, while charges against his associates were dismissed.

A more detailed investigation of the seized items revealed the truly dangerous nature of von Rosen's mission, as the sugar cubes had been infused with anthrax and a solution possibly containing typhus. This discovery caused panic among Norwegian authorities and allies, leading to drastic measures. In response, Norwegian police broke the diplomatic seals on the luggage of an envoy from the German Foreign Ministry, revealing a large stockpile of explosives and biological agents intended for sabotage and supporting the revolutionaries in Finland. In 1997, the vials were found in a police museum in Trondheim, Norway, and further analysis showed fragments of anthrax *Bacillus* [19]. The results of the potential attacks remain uncertain because the remote locations, widespread occurrence of anthrax, and subsequent turmoil in Tsarist Russia limit the availability of sources.

Biosabotage on neutral countries

One of the initial plans, which was ultimately rejected by Berlin, involved contaminating Portuguese rivers with vibrio cholerae [15,20]. This illustrates the German army's willingness to use disease as a weapon of war, although the politicians of the time, to their credit, denied it. Another, this time, verified case of biological sabotage was the contamination of Romanian sheep exported to Russia with *Bacillus anthracis*. However, the German effort was not implemented in time as Romania declared war on Austria and Germany on August 27, 1916, the same day the cultures arrived at the espionage team in Bucharest and were buried in the garden of the German mission. A month later, the cultures were discovered along with 50 bundles of trinitrotoluene (TNT) and identified as anthrax and *B. mallei* by Dr. Victor Babes, a professor at the Institute of Microbiology and Bacteriology in Bucharest [21].

The following year, French forces on the Western Front issued a general warning that an agent had been captured with vials of B. mallei and instructions to infect horses or their feed. After the armistice, it was found that the German consul in Zurich was organizing the shipment of cholera cultures to Italy through agents. Also, Berlin approved the use of *B. anthracis* and *B. mallei* against Argentine sheep, cattle, and horses, respectively, which were supplying Britain and the Indian Army. Anthrax was typically placed inside sugar cubes that were transported by submarine to the envoy in Buenos Aires. As a result of the attack, 200 mules died in 1917 and 1918. It is less certain whether the deaths of cattle and equines from anthrax and glanders on ships arriving in Bordeaux from Argentina in 1916 can be attributed to the Germans [15].

Waging biowarfare in the USA

**Figure 1:**
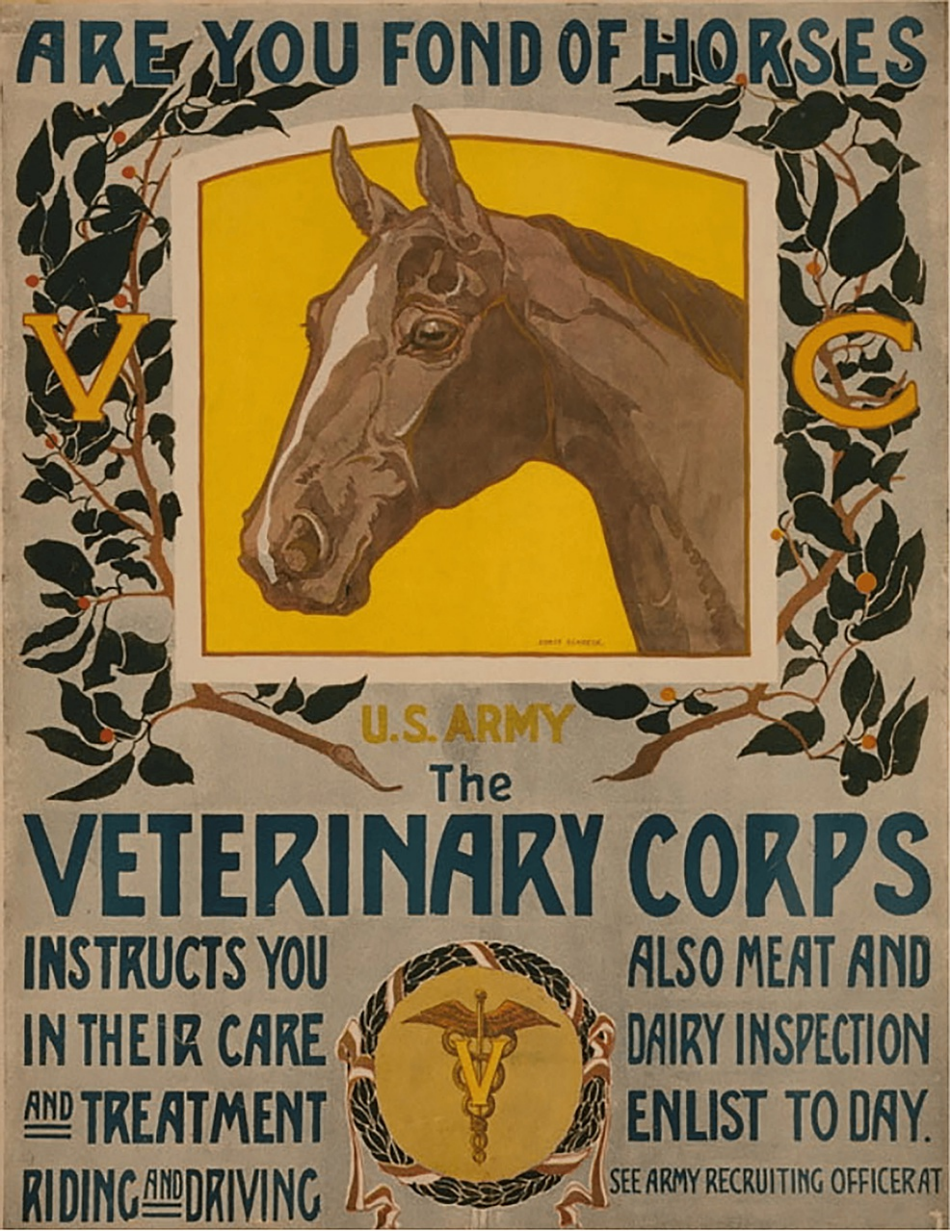
A World War I poster featuring a portrait of a horse and insignia of the Army Veterinary Corps Rights advisory: No known restrictions on publication. For information, see 'World War I Posters' (https://lccn.loc.gov/2002722430l).

In the USA, Franz von Papen (1879-1969) [22], the military attaché in Washington and later Chancellor of Germany, had been practicing covert actions among other sabotage activities with the aforementioned pathogens in 1915 and could be responsible for infections of animals in ports. His unsuccessful activities were continuously monitored by British intelligence and the Americans [23]. Also, Anton Dilger (1884-1818), son of a German immigrant and a hero of the American Civil War, was sent to Germany at the age of nine, where he earned his medical degree in 1912. With the onset of the war, he served in German military hospitals on the battlefield. Seeing his relatives injured and dead in the conflict accelerated his shift to a pro-German and anti-Ally stance. On September 29, 1915, when he left Germany for Washington, he brought a small portfolio filled with vials containing strains of anthrax and glanders [24]. Along with his brother Karl, a former brewer, they set up a lab in a private house in Maryland, where they cultivated these bacteria for several months. With the help of companions and handlers, they secretly introduced anthrax into horses, while the glanders agent was embedded in their nasal cavities or poured into their water. These actions were carried out at various East Coast ports, such as Norfolk, New York, and Baltimore. The Dilgers continued to operate their lab until January 29, 1916, when Anton suddenly returned to Germany after learning that the New York police had begun asking questions about their activities, and guards were placed on the stabled equines. This vigilance managed to prevent a widespread biological attack, and Anton Dilger escaped to Germany, where he was honored with the Iron Cross in January 1918 in recognition of his work. However, he soon died of the Spanish flu in Madrid the same year. Dilger's actions have been well documented, as they caused a great sensation in the USA. He has been referred to as "the man with the secret mission to bring the Great War to the USA" (Figure 1) [25,26].

The moral constraints of waging biowarfare and the end of WW I

Some less reliable post-war French sources refer to other alleged German secret biowarfare activities, such as an attempt by a German agent to spread smallpox in Saint Petersburg in 1915, the arrest of another German agent with similar intentions in Russia in 1916, and a supposed successful infection of 4,500 mules with glanders in Mesopotamia in 1917 [23], but the above seem not to be substantiated by any evidence. There were also reports of the use of the Colorado Potato Beetle (*Leptinotarsa decemlineata*) from both belligerents, a beetle that massively destroys potato crops, but these remain equally unproven [27]. For example, in October 1917, the British received information from a French source that "the enemy had infected a large number of rats with plague and intended to release them in the United Kingdom from submarines or airplanes." In September 1916, Oberstabsarzt Winter, an officer of the 21st military corps, proposed using Zeppelins to drop plague bombs on English ports. In response to the proposal, the German chief medical officer replied that "if we take this step, we will no longer deserve to exist as a nation" [18]. 

At the end of the war, the German General Staff deliberately destroyed most of the documents related to the biological sabotage program. However, studies of the sabotage campaign conclude that the bacteriological laboratory of the German Military Academy in Berlin was the likely source of the glanders and anthrax cultures used during the war. Germany’s engagement in biological warfare, utilized as a method of sabotage, was instrumental in setting the stage for the extensive development of offensive biological warfare programs among the major world powers of that era. Notably, this included the Soviet Union, which found itself encircled by nations that were antagonistic toward its communist ideology [18]. This marked a pivotal moment in military history: for the first time, the application of scientific advancements enabled the creation of formidable biological weapons against which there was minimal or virtually no effective defense [14]. 

The effect of bioattacks

Despite the sophisticated development of biological weapons during WWI, their effectiveness was limited due to several key factors. This restrained use was affected by moral constraints and the inherent risks these weapons posed. Moreover, the use of biological agents against enemies could potentially backfire, with the agents returning to German territories either through environmental factors or retaliatory measures by adversaries. This fear of mutual destruction added a layer of restraint to utilizing biological warfare to its full lethal potential. Additionally, open-field testing of biological agents had not been properly initiated, leaving a gap in understanding the actual impact and spread of these pathogens in combat scenarios [28]. The lack of comprehensive field trials resulted in uncertainties about the behavior of pathogens outside the laboratory, reducing confidence in their strategic deployment. As later noted in the reports by prominent Soviet biowarfare scientist Yakov Moisseevich Fishman (1887-1962), for a biowarfare program to be effective, the following three stages are necessary: laboratory, chamber, and open field.

Open-field testing requires finding an isolated location that meets the technical objectives of the experiments and ensures secrecy and safety. Fishman mentions the mandatory start of experiments with either non-lethal or infectious bacteria to characterize a danger zone and to study the impacts and bacterial populations in the environment. The above methods and experimental equipment were acquired by the Germans post-war under a renewed program [18,29]. The case studies, such as the limited contamination of supplies meant for enemy nations and the sporadic attempts to infect livestock via small-scale attacks, demonstrate these limitations. While these efforts indicate a capability to manufacture and deploy biological agents, the actual application was cautious and minimized, simulating mostly open-field testing stages.

## Conclusions

The German program was significant for several reasons. First, it was the first time that biological warfare was used aggressively in an organized manner. Second, it was based on scientific understanding, and third, it demonstrated how bacteria could be used covertly to change the course of the war. The program continued secretly until 1933, and there were tests of biological weapons by the Germans, mainly with anthrax and glanders. Although it was a catalyst for the creation of several other biological programs, with the rise of Adolf Hitler to power, the offensive biological programs were discontinued. Throughout the war, Germany's use of biological warfare aimed primarily at livestock illustrated a strategic application of medical science in military operations, but the pathogens used could pose great risk to nearby residing populations. Furthermore, the German biowarfare effort illustrates the critical role of medical professionals in wartime, not only in healing but in preventing misuse of medical knowledge. It highlights the necessity for ongoing ethical education and strict adherence to medical ethics among health professionals.

In conclusion, the medical consequences of Germany's biowarfare efforts during WWI laid the groundwork for the subsequent misuse of medical science in ways that would be significantly intensified during the Cold War. In summary, the biological attacks during WWI were not as effective as potentially envisioned due to their limited scale, ethical and self-preservation concerns among the Germans, and the lack of thorough open-field testing. These factors combined to make the bioattacks more of an exploration rather than a full-scale operational warfare tactic. Ultimately, the use of biological weapons in WWI highlighted the early stage of modern bio-warfare, marked by restraint and the significant risks it posed to both the user and the target.
